# Double Trouble Co-Infections: Understanding the Correlation Between COVID-19 and HIV Viruses

**DOI:** 10.7759/cureus.38678

**Published:** 2023-05-07

**Authors:** Sassi Ashraf Ali Abbasi, Tarika Noor, Maneeth Mylavarapu, Monika Sahotra, Hunmble A Bashir, Rakshita Ramesh Bhat, Urmi Jindal, Uzma Amin, Anushree V, Humza F Siddiqui

**Affiliations:** 1 Primary Care, Sindh Institute of Urology and Transplantation, Karachi, PAK; 2 Department of Medicine, Government Medical College, Patiala, Ludhiana, IND; 3 Department of Public Health, Adelphi University, Garden City, USA; 4 Department of Medicine, Bukovinian State Medical University, Chernivtsi, UKR; 5 Forensic Medicine, Combined Military Hospital (CMH) Lahore Medical College and Institute of Dentistry, Lahore, PAK; 6 Medical Oncology, Mangalore Institute of Oncology, Mangalore, IND; 7 Internal Medicine, Bangalore Medical College and Research Institute, Bangalore, IND; 8 Department of Medicine, Karamshi Jethabhai Somaiya Medical College, Mumbai, IND; 9 Pathology, Rawalpindi Medical University, Rawalpindi, PAK; 10 Department of Medicine, Jagadguru Jayadeva Murugarajendra (JJM) Medical College, Davangere, IND; 11 Department of Medicine, Jinnah Sindh Medical University, Karachi, PAK

**Keywords:** sars-cov-2 infection, anti-retroviral therapy, pandemic, people living with hiv, hiv, covid-2019

## Abstract

A global outbreak of coronavirus disease 2019 (COVID-19), an infectious disease caused by the severe acute respiratory syndrome coronavirus 2 (SARS-CoV-2), mounted a substantial threat to public health worldwide. It initially emerged as a mere outbreak in Wuhan, China, in December 2019 and quickly engulfed the entire world, evolving into a global pandemic, consuming millions of lives and leaving a catastrophic effect on our lives in ways unimaginable. The entire healthcare system was significantly impacted and HIV healthcare was not spared. In this article, we reviewed the effect of HIV on COVID-19 disease and the ramifications of the recent COVID-19 pandemic over HIV management strategies.

Our review highlights that contrary to the instinctive belief that HIV should render patients susceptible to COVID-19 infection, the studies depicted mixed results, although comorbidities and other confounders greatly affected the results. Few studies showed a higher rate of in-hospital mortality due to COVID-19 among HIV patients; however, the use of antiretroviral therapy had no consequential effect. COVID-19 vaccination was deemed safe among HIV patients in general. The recent pandemic can destabilize the HIV epidemic control as it hugely impacted access to care and preventive services and led to a marked reduction in HIV testing.

The collision of these two disastrous pandemics warrants the need to materialize rigorous epidemiological measures and health policies, but most importantly, brisk research in prevention strategies to mitigate the combined burden of the two viruses and to battle similar future pandemics.

## Introduction and background

A global outbreak of coronavirus disease 2019 (COVID-19), an infectious disease caused by the severe acute respiratory syndrome coronavirus 2 (SARS-CoV-2), mounted a substantial threat to public health throughout the world. The first cases were detected in China in December 2019, with the virus spreading rapidly to other countries globally. A public health emergency of international concern was declared on 30 January 2020 and was characterized as an outbreak of a pandemic on 11 March 2020 [1]. The patients usually present with severe acute respiratory syndrome, dyspnea and pneumonia, hyperinflammatory response, vascular damage, angiogenesis, and thrombosis. Several organ systems, including respiratory, cardiovascular, and neurological, can be involved. To date, no specific treatment for SARS-CoV-2 infection provide promising results exists. Currently, antiviral agents, inflammatory inhibitors/antirheumatic drugs, low molecular weight heparins, plasma, and hyperimmune globulins are used for the treatment of infection [2]. Vaccines brought hope to the world, on the other hand, the mutations in the virus brought fear. The diagnosis of COVID-19 became more challenging with the emerging variants of the virus [3]. Nucleic acid-based tests or antigen detection tests are utilized for the diagnosis of the disease [4]. Currently, reverse transcription-polymerase chain reaction (RT-PCR) is considered the gold standard for qualitative and quantitative detection of viral nucleic acid [5]. Different techniques can be used to enhance the detection efficacy, such as a chest X-ray [6]. Patients end up either with recovery or death. According to the latest update by WHO, as of 7 March 2023, approximately more than 750 million cases of COVID-19 have been confirmed and around 7 million deaths have been reported across the world due to SARS-CoV-2 infection as of March 2023 [7].

Human immunodeficiency virus types 1 and 2 (HIV-1 and HIV-2) are two lentiviruses that cause acquired immunodeficiency syndrome (AIDS) in humans. Here, we discuss the history of these viruses, their development, and the events that contributed to the AIDS pandemic. Simian immunodeficiency viruses (SIVs), which normally infect African monkeys, were the cause of many cross-species transmissions that gave rise to both HIVs [8]. HIV lentivirus interacts with a wide range of body cells to produce AIDS while evading the host immune system's defenses. HIV is typically passed from infected women to their newborn babies through genital and blood fluids [9]. Blood transfusions, organ/tissue transplants, breastfeeding, needle prick, and sexual contact all transmit the HIV virus from one person to another [10]. HIV interacts with several recently discovered cellular receptors in addition to the cluster of differentiation 4 (CD4) protein on cell surfaces during the stages of infection and HIV enters the cell upon virus-cell fusion. Many intracellular processes regulate the relative expression of viral regulatory and auxiliary genes after the virus houses the cell, resulting in productive or latent infection. Further advanced studies are needed to develop a cure for this deadly global disease [9]. Perhaps the most significant public health catastrophe of our time, the HIV-1 pandemic is a complicated mash-up of several diseases that have spread inside and across nations and regions of the world. Alternate strategies for prevention have notably increased and curtailed the global burden. However, despite the sophistication of our knowledge of pathophysiology and transmission dynamics, a definitive cure or protective vaccination is still unattainable [11].

The COVID-19 pandemic has caused a unique public health emergency worldwide [12]. Starting from January-February 2020, numerous outbreaks led to a massive global public health response, with schools, public gatherings, and non-essential service providers being closed down through city, state, and country-wide measures. Medical care was also restricted to essential functions only. In response to the COVID-19 epidemic, HIV clinics were forced to cancel non-urgent visits, transition in-person visits to remote telephone visits when necessary, redirect HIV and infectious diseases providers to inpatient care, public health, occupational health, or logistical duties, and reduce routine laboratory testing and social services for people living with HIV (PLWH) [13]. There is a lack of information on how COVID-19 affects people living with HIV (PLWH), who are considered an immunocompromised population. Currently, there are around 38 million PLWH globally, with 690,000 HIV-related deaths in 2019 [14]. The World Health Organization (WHO) estimates that only 75% of PLWHs were on antiretroviral therapy (ART) worldwide in 2021 [15], and only 60% were aware of their HIV status [16]. This means that PLWH who are not on ART or whose disease is not well controlled may have a higher risk of contracting COVID-19 due to their compromised immune systems. They may also be at a higher risk of developing severe symptoms and even death if infected with COVID-19. However, there is limited information available on the treatment and outcomes of SARS-CoV-2 infection among PLWH. Two studies in New York found that outcomes of people with and without HIV who were hospitalized for COVID-19 were similar, even after accounting for various confounding and mediating factors [17-19].

In the United States, increasing age, obesity, diabetes, and African American or Hispanic race have all been linked to an increased risk of contracting COVID-19 and experiencing severe disease, but HIV and other factors have not been clearly linked to this risk [20-25]. Moreover, individuals with HIV frequently have associated risk factors for coronavirus, including male gender, African American or Hispanic ethnicity, more seasoned, having comorbid medical conditions, and being a smoker, at rates higher than the general population [26-33]. HIV patients continue to have higher levels of inflammation, which is a major contributor to the development of severe respiratory disease, thromboembolic disease, and other COVID-19-related adverse outcomes [34,35]. Effective resource management strategies are needed by healthcare systems to address the additional epidemiological and physiological risks posed by COVID-19 to PLWH. Anti-retroviral (ARV) multi-month dispensing policies and telemedicine platforms are being utilized in several countries to minimize supply disruptions and reduce COVID-19 exposure. A multidisciplinary approach is critical for PLWH to maintain their physical and mental well-being during the pandemic. [17,36,37].

COVID-19 has been a global mammoth catastrophe that momentarily overlapped with the HIV calamity and hence it is of utmost clinical significance to periodically review the correlation between the two viruses. Subsequently, this meticulous literature review aims at presenting the available data regarding the susceptibility and outcomes of coronavirus among HIV infected population, the role of anti-retroviral therapy, and the impact of the current COVID-19 pandemic on the ongoing HIV pandemic to help clinicians provide better optimal care to PLWH and assist public health organizations in understanding and be prepared for future medical adversities.

## Review

Susceptibility to SARS-CoV-2 infection among HIV patients

In the study on how HIV infection affected the quality and epitope specificity of SARS-CoV-2 T cell responses, conducted during the first and second waves of the COVID-19 pandemic in South Africa, researchers found that unsuppressed HIV infection significantly reduces T cell responses to SARS-CoV-2 infection. When compared to HIV-infected individuals who were viremic, HIV-seronegative individuals showed considerably higher CD4+ T cell responses against the Spike protein, a large surface transmembrane protein. T cell activation was negatively connected with CD4+ T cell responses (CD4 r=0.7, p=0.04), although absolute CD4 count positively correlated with SARS-CoV-2-specific CD4+ and CD8+ T cell responses (CD4 r=0.5, p=0.03; CD8 r=0.5, p=0.001). They also observed a reduced T cell cross-recognition between the two waves, which was particularly prominent in patients with unsuppressed HIV infection [38]. These findings underscore the vulnerability of PLWH to new SARS-CoV-2 variants of concern and may partially explain the higher susceptibility of PLWH to severe COVID-19.

COVID-19 is a pandemic caused by a new strain of coronavirus called SARS-CoV-2. The pathology behind the causation and severity of the disease is believed to be due to a cytokine storm caused by the host’s immune system. HIV is a virus that targets the body’s immune system and leads to immune deficiency. This depletion in the immune system is believed to decrease the chances of cytokine storm or the severity of the storm, rendering HIV-infected patients less susceptible to COVID-19 infection. Two studies in Spain initially suggested that the incidence of COVID-19 infection among people living with HIV is lower compared to the general population. A prospective cohort study in Barcelona with 5,683 PLWH on ART, found that 53 (0.9%, 95% CI - 0.7% - 1.3%) developed confirmed or suspected COVID-19 infection during the time of the study and the standardized incident rate ratio of confirmed infection was 38% (95% confidence interval 27%−52%) in comparison to the general population [39]. In a prospective cohort study conducted among HIV clinics in 60 hospitals that treat 77,590 PLWH in the Madrid region, the researchers observed 236 COVID-19 infections among PLWH. This study also estimated that the age- and sex-standardized risk of confirmed COVID-19 infection among PLWH on ART is lower compared to the general population (30 per 10,000 among PLWH vs 41.7 per 10,000 among the general population) [40].

There are studies that got contrasting results to the hypothesis. In a study conducted in France which included 77 COVID-19 diagnoses among PLWH the estimated attack rates of COVID-19 among PLWH and the general population were similar, and with the help of multivariable analysis, it was found that HIV was not associated with COVID-19 [41]. A small population cohort study in Wuhan, China, found that standardized incidence rates of COVID-19 among 6,001 PLWH (0.38%, 95% confidence interval 0.24 - 0.53%) and the general population (0.45%, 95% confidence interval 0.45% - 0.46%) are similar [42]. Few studies found HIV infection increases the susceptibility to COVID-19 infections. The Veterans Aging Cohort Study (VACS), with 253 PLWH co-infected with COVID-19, found that testing rates among PLWH were higher than those without HIV, but the test positivity rates remained relatively similar, with 9.7% of PLWH vs 10.1% of HIV-uninfected veterans being diagnosed with COVID-19 in between February and April 2020 [43]. A population-based study in San Francisco found PLWH were more likely to test positive for COVID-19 during the first six months of community spread. The positivity rate among PLWH was 4.5% (193/4252) as compared to 3.5% (9626/272,555) among people without HIV [44].

Effect of HIV on the severity of COVID-19 infection outcomes

Early pandemic data did not indicate large rates of severe COVID-19 infections among people living with HIV (PLWH). Nevertheless, the majority of these data were from single-center studies, hospitalized cohorts, or observational case series and were thus subject to biases including the possibility of PLWH being hospitalized out of caution regardless of clinical state [45]. A cohort of 3,460,932 patients covered by the public sector health care system in the Western Cape Province, South Africa, includes 540,552 PLWH, or 3978 instances of COVID-19, making it the biggest published population-based cohort to date. After adjusting for confounders of age and sex, PLWH had a higher risk of dying from COVID-19 (adjusted Hazard Ratio {aHR} 2.14; 95% CI: 1.70-2.70) than people without HIV. There were some remarkable characteristics of this cohort, such as the high rates of co-occurring TB and uncontrolled diabetes and the low incidence of proven virologic suppression during the previous 15 months (45%) [46]. However, the study conducted using the Open SAFELY platform from the UK primary care clinic system, which comprised 27,480 PLWH in a population of 17,282,905 people, also showed higher mortality. After controlling for age and sex, PLWH had a greater risk of COVID-19 mortality than people without HIV (aHR 2.59 {95% CI 1.74-3.84}) [30]. There was an elevated adjusted odds of mortality (AOR 1.29 {95% CI 1.16-1.44}) among PLWH in the US National COVID Cohort Collaboration, which comprised 13,170 cases with COVID-19 [47]. The death risk was also greater among 2988 PLWH diagnosed with COVID-19 in New York State compared to those who were HIV-negative (standardized RR, 1.23 {95% CI, 1.07-1.40}) [48]. The adjusted risk of severe or fatal COVID-19 was 1.30-fold higher among PLWH (95% CI: 1.24-1.36) than those without HIV within the WHO Global Clinical Platform, which contained hospitalization data on 15,522 PLWH from 24 countries: however, the major proportion of the data came from South Africa [49].

The adjusted HR meta-analysis added two investigations (one from South Africa and one multicentric) in addition to the 23 studies that were included in the unadjusted risk ratio meta-analysis. There was no correlation between HIV and mortality from COVID-19 in unadjusted pooled analysis (OR: 0.81; 95% CI 0.47; 1.41, 23 studies). HIV, however, was linked to a higher risk of mortality in analyses controlling for age and sex (hazard ratio 1.76, 95% CI 1.31-2.35; 2 studies). Even after stratification by the study's country of recruitment, a meta-analysis of 13 studies that included 13,016 HIV-infected individuals with COVID-19 and 1,744,014 HIV-uninfected individuals with COVID-19 reveals that HIV does not increase the likelihood of having severe COVID-19 (odds ratio {OR}: 1.28; 95% CI 0.77-2.13). HIV-positive COVID-19 participants were more likely to be admitted to the hospital than HIV-negative individuals, according to the meta-analysis of six trials (OR: 1.49; 95% CI 1.01-2.21) [50].

Various studies discovered that HIV patients with low CD4 counts and compromised immune systems who are in the advanced stages of the illness (stages 3 or 4) exhibit less severe COVID-19 symptoms. Yet, several investigations produced contentious findings that run counter to our major theory. The lack of existing literature and inconsistent evidence, which hindered our capacity to address and persuasively defend our key premise, were the main causes of these discordant outcomes. This may also be related to concurrent symptoms and underlying comorbidities that develop at later stages of HIV infections and might confuse and obfuscate the normal presentation of COVID-19 in such individuals. Despite these contradicting findings, the majority of literature reflects COVID-19 symptoms in HIV patients to be minimal or nonexistent, especially in those with advanced HIV illness. In addition, few studies discovered an unexpectedly high percentage of recovery in these individuals following COVID-19 infection, defying the conventional wisdom that immunocompromised people had a greater risk of morbidity and mortality. Fever, coughing, headaches, shortness of breath, fatigue, loss of taste or smell, and gastrointestinal symptoms including diarrhea, anorexia, nausea, and abdominal discomfort are typical symptoms of COVID-19, and many of them are brought on by the cytokine storm that the host's immune system produces. Immune insufficiency is brought on by the human immunodeficiency virus, which attacks the body's defense mechanisms. The intensity of immune system responses, such as cytokine storms, and consequently the accompanying symptoms, can be reduced by this immunological deficit. As the major fatal disease in COVID-19 patients is caused by the cytokine storm, which then results in multiorgan failure and death, this may explain the milder symptoms, reduced morbidity, and decreased mortality among HIV-positive individuals infected with COVID-19. Several of the studies included in the current evaluation provided evidence in favor of the concept, while some investigations also produced incongruous results. Future research should study the potential underlying reasons for such an observation in more detail in light of the conflicting results [51].

All HIV-positive individuals in the study by Calza et al. who had a CD4 level below 258 made a full recovery [52]. Additionally, the patients in Kumar et al. and Patel and Pella's study had a CD4 level lower than 500 and all made a full recovery [53,54]. Mondi et al. obtained similar results [55]. There are studies that found contrasting results to this hypothesis. Karmen‐Tuohy et al. reported that the mortality rate was higher (28%) in the patients who had a median CD4 count < 500 [56]. Similarly, in the study by Blanco et al., every patient with a CD4 count > 500 got completely cured, but one of the two patients with a CD4 count < 500 had prolonged hospitalization due to the severity of the illness that may have been due to comorbidities as reported by the authors [57]. The findings of the Ruan et al, study were completely against the hypothesis, all the patients with CD4 count > 500 experienced moderate to severe clinical outcomes, where-in all the patients with CD4 count < 500 had severe clinical outcomes [58].

The relationship between COVID-19 and HIV is not linear and may be influenced by the differential impact of various comorbidities and social determinants of health. It can be said that the interplay of various social determinants of health may have a huge role in PLWH mediating the risk of exposure to COVID-19 infection rather than the mere previous HIV-positive status. SARS-CoV-2 infection contraction may be higher among PLWH due to increased rates of chronic lung disease and lung-damaging habits including smoking, inhaling drugs, and excessive alcohol consumption. PLWHs have higher rates of homelessness and unstable housing situation (crowded housing) compared to the general population and increased stay time in homeless shelters and congregate living settings. This may have a negative impact on their ability and chances to distance themselves or effectively quarantine socially [13].

Prognosis of COVID-19 in HIV-infected population

The prognosis of COVID-19 in PLWH is variable according to the evidence found globally and is attributed to different study designs, whether the PLWH are taking consistent ART, and the presence of comorbidities in PLWH. A study performed in New York City identified 88 PLWH hospitalized with laboratory-confirmed COVID-19 compared to a control group of uninfected individuals. Patients did not differ significantly by HIV status by age, sex, or race/ethnicity due to the matching algorithm. There was no difference in COVID-19 severity on admission by HIV status. However, PLWH had greater proportions of smoking and comorbid illness than uninfected comparators [18]. In contrast, another cohort study conducted in New York City showed that COVID-19 patients with HIV infection had a higher prevalence of hypertension, diabetes mellitus, dyslipidemia, heart failure, and chronic kidney disease compared to COVID-19 patients without HIV infection [59].

A well-resourced systematic review and meta-analysis of 22 studies, involving more than 20 million people living with HIV (PLWH) done in skilled tertiary setup showed that low CD4 count, high AIDS rate, and a high prevalence of multiple co-morbidities among PLWH with COVID-19 disease, seem to have a key role in the high COVID-19 mortality. A study done in Cape Town, South Africa compared the rate of hospitalization in different stages of HIV disease. Their study stated that the rate of hospitalization was 69% higher in stage 4 (CD4 less than 200 cells/ml) and 29 % higher in stage 2 (CD4 count was 200-499) compared to stage 1 (CD4 counts > 500 cells/ml) [60].

The data from the International Severe Acute Respiratory and Emerging Infection Consortium (ISARIC) database found that hospitalized PLWH with COVID-19 had a 63% higher mortality than their HIV-negative counterparts, after adjusting for age, ethnicity, comorbidities, and disease severity when they presented to the hospital. Similarly, results from the Open SAFELY dataset found a more than two-fold increase in mortality. Notably, both studies lack data on HIV-related parameters such as antiretroviral therapy (ART) use, HIV-1 viral load, CD4 T cell count, and prior opportunistic infections, all of which are important confounders [47,61].

HIV is a disease of a chronic inflammatory state, and elevated interleukin (IL)-6 levels are associated with older age and higher BMI hence higher viral replication and low nadir CD4+ T cell count could be associated with the poor outcome when infected with COVID-19 [62]. One study linked the role of liver fibrosis and underlying advanced liver disease as a significant risk factor of mortality that was associated with chronic viral hepatitis B and or C in HIV patients. Among the Spanish population, younger age and lack of comorbidities were linked to better prognosis in PLHIV [63]. Although some scholars have speculated that antiretroviral drugs may favor HIV patients due to their activity against SARS-CoV-2 and other coronaviruses, there has been no evidence that HIV patients receiving certain antiretroviral drugs have an altered risk of COVID-19 infection and severity [64,65]. A study found that older age, late diagnosis, low CD4 cell count, and treatment-naive status were potential determinants of COVID-19 incidence among HIV patients. Similarly, several studies performed globally confirmed that advanced age and preexisting comorbidities, such as hypertension and diabetes, are associated with unfavorable outcomes and increased mortality from COVID-19 [66,67].

Effect of anti-retroviral therapy (ART) on COVID-19

Various anti-retroviral drugs act against enzymes that are involved in the replication of the coronavirus. Tenofovir which is a nucleotide analog that binds to the RNA polymerase enzyme can be used to treat COVID-19 patients. One of the first few studies that examined the efficacy of various readily available antivirals was by Elfiky et al. The study concluded that Tenofovir, Ribavirin, Remdesivir, Sofosbuvir, and Galidesivir showed efficacy against RNA dependent RNA polymerase enzyme of the SARS-CoV-2 strain. Since these drugs were extensively tested and already FDA-approved, no further studies to assess their efficacy and toxicity profiles were deemed necessary [68]. As to whether suppressive ART has any direct effect on disease susceptibility and severity, studies have shown varying results. While it might be reasonable to assume that a higher CD4 count and a suppressed viral load might be associated with a better prognosis, there are also several other factors that determine disease outcomes. Few studies showed that HIV and active tuberculosis were independently associated with increased mortality due to COVID-19. Tenofovir disoproxil fumarate (TDF) has also been associated with fewer COVID-19 deaths [46]. In an analysis among people living with HIV and pre-exposure prophylaxis users from a single center in France, tenofovir use was not associated with a risk of COVID-19 infection or clinical outcome [41]. Another study conducted across 60 clinics in Spain reported that TDF-based ART, but not tenofovir alafenamide (TAF) was associated with lower rates of both COVID-19 diagnosis and hospitalization. Patients receiving tenofovir disoproxil fumarate/emtricitabine (TDF/FTC) had the least risk of COVID-19 diagnosis and hospitalization [40]. Guo and colleagues reported that 199 HIV-infected persons taking ritonavir-boosted lopinavir or integrase strand transfer inhibitors (INSTIs) had no cases of COVID-19, whereas eight of 947 patients taking nucleoside reverse transcriptase inhibitors (NRTIs) plus non-nucleoside reverse transcriptase inhibitors (NNRTIs) were infected [69]. A single-center prospective cohort study conducted in Spain compared baseline characteristics of HIV-infected individuals with and without COVID-19. The two groups did not vary much in age distribution, nadir CD4 cell counts, and the proportion of individuals on ART. HIV-infected individuals with COVID-19 had a higher mean body mass index (BMI) than those who did not have COVID-19 and a higher prevalence of chronic comorbidities. As for ART, a significantly higher proportion of those with COVID-19 received tenofovir, either as tenofovir alafenamide or TDF, before the diagnosis of COVID-19 than those without COVID-19 [70].

Major Protease (MPro) enzyme is necessary for viral replication in SARS-CoV-2 and protease inhibitors can help arrest viral replication. Lopinavir, ritonavir, and other protease inhibitors target the major protease enzyme of SARS-CoV-2 [71]. Atazanavir, also a protease inhibitor docks in the active site of SARS-CoV-2 major protease. It can be used alone or in combination with ritonavir. This combination also decreases virus-induced elevation of levels of IL-6 and tumor necrosis factor-alpha (TNF-α) [72]. Ritonavir in combination with molnupiravir (nucleoside analog) and nirmatrelvir (protease inhibitor) has received emergency use authorization for the Omicron SARS-CoV-2 virus variant [73].

COVID-19 vaccination in the HIV-positive population

Viral vector and mRNA are the two types of vaccines for COVID-19 infection that are in use. But the questions to contemplate are: Are these vaccines safe for people living with HIV? Do they provide the same results in HIV-positive people and others? Are there any risk factors of COVID-19 vaccination in HIV-positive patients?

The majority of the published literature suggests that both types of vaccines are considered to be safe in HIV-positive patients. Even vaccines with the viral vector are shown to be harmless in one of the most recently published studies. Most of the clinical trials that have been conducted showed that these vaccines do not interfere with the effectiveness of anti-retroviral treatment given to HIV patients. No significant reactions are found between the ART and COVID-19 vaccinations that can lead to any troublesome outcome. Centre disease control and Prevention (CDC) recommends that HIV-positive individuals should be administered the COVID-19 vaccination until and unless there are some severe immediate or delayed allergic reactions. According to the British HIV Society, the protection provided by the vaccine is comparatively lower in people with a CD4+ count of less than 100. However, there are no such studies suggesting that the vaccine should not be administered in people with low CD4+ count. Every patient irrespective of their CD4+ count should receive the vaccine [74].

Ample studies were conducted to check the immunogenicity. One of the studies showed that the mean anti-RBD immunoglobulin (Ig)G responses were almost similar between the HIV-negative control group and the HIV+ group. Nevertheless, the responses were found to be remarkably lower in people with a CD4+ count of less than 250. However, HIV-positive people with a CD4+ count of less than 250 have a weaker response suggesting the need for a booster dose in this subgroup. This study was conducted after the first dose of an mRNA anti-SARS-CoV-2 vaccine was administered to HIV patients [75]. Apart from this, a study performed to know the status of HIV RNA levels following SARS-CoV-2 vaccination, revealed no significant change in HIV-RNA levels [76].

A cohort study conducted in the United States revealed that the incidence rate of breakthrough infections was higher in people living with HIV after complete vaccination as compared to people living without HIV. The incidence of breakthrough infection differed by the type of vaccine and pharmaceutical brand. There was no association with the HIV viral load but the risk of breakthrough infection decreased with the increase in the CD4+ count [77]. Another cohort was conducted to know the efficacy of the BNT162b2 mRNA vaccine in HIV-positive patients. It was found that the RBD-IgG antibody levels were lower in HIV-positive individuals as compared to immunocompetent individuals. However, the BNT162b2 mRNA vaccine was found to be immunogenic and safe in people living with HIV [78].

A crucial barrier was that despite having the knowledge of the elevated risk of getting infected with COVID-19, many people were hesitant towards vaccination. But the fact that the vaccination was compulsory led to a higher compliance rate [79]. A study has shown that over half of HIV-positive people were unsure about the efficacy of COVID-19 vaccine [80]. A cohort study was conducted to know the efficacy of the mRNA COVID-19 vaccine in HIV-positive patients which was found to be high with no significant difference between positive and negative control groups [81]. However, in a cross-sectional study in China, it was found that HIV-positive men having sex with men (MHSM) had a high willingness to receive COVID-19 vaccination [82]. The other challenges were the global access and distribution of the vaccine; however, the vaccine was successful in bringing hope to the world [83]. According to the latest report of WHO, as of March 2023, a total of 13,299,166,046 vaccine doses have been administered worldwide [7].

Impact of COVID-19 on the mental health of HIV-positive adults

The initial reports of COVID-19 indicated that the disease could lead to severe respiratory distress and illness, particularly in high-risk groups, such as those aged 60 years and above, people with underlying medical conditions or chronic illnesses, and those with weakened immune systems (including individuals with HIV, chronic lung disease, asthma, or heart conditions) [84-88]. Therefore, people living with HIV (PLWH) are thought to be at an increased risk of experiencing negative outcomes as a result of COVID-19 [58,89,90]. Although recent studies that retrospectively analyzed hospitalized PLWH with COVID-19 have not shown higher rates of infection or severity among PLWH, there is a shortage of literature on this topic and a lack of long-term follow-up [91-93].

Certain groups of people, such as older adults, individuals with obesity, compromised immune systems, and other medical conditions, are more likely to experience severe illness from COVID-19 [94-97]. Among these groups, older women with HIV are particularly vulnerable [46,49]. As per the reports in 2018, in the United States incidence of HIV increased among people aged 50 and above. Similarly, women over 55 constituted a significant proportion of 17% of newly reported cases of HIV in 2021 [98]. Women with HIV already face significant challenges related to gender, age, and other medical conditions, which may be exacerbated by the COVID-19 pandemic. Communities of color have been disproportionately affected by COVID-19, which may be due to economic disparities and limited access to healthcare. This puts women from underserved communities and/or communities of color, who are the majority of older women with HIV in the US, at a higher risk of experiencing major disruptions in their lives due to COVID-19. In the few surveys conducted participants reported experiencing increased anxiety and depression during the COVID-19 pandemic. Daily activities such as medical appointments became sources of anxiety [99-102]. Because of physiological and mechanical alterations during pregnancy, pregnant women and their fetuses are at increased risk for COVID-19 infection [103,104]. The virus can have social and emotional effects on pregnant and postpartum women who may be separated from their families and communities due to quarantine measures [105,106]. Reducing prenatal discomfort for pregnant women and their babies is important because the long-term psychological and neurological impacts can be significant. It's crucial to take into account the mental health consequences of the COVID-19 pandemic on expectant mothers, to prevent the development of severe mental disorders after giving birth [103,104]. During the COVID-19 pandemic, pregnant women's anxiety and depression symptoms were found to be higher than those of men [107,108].

Ade-Ojo et al.’s study found that during the COVID-19 pandemic, pregnant HIV-positive women experienced significantly higher levels of depression and anxiety compared to HIV-negative pregnant women [109]. Almost half of the HIV-positive pregnant women in the study had major depressive disorder, with a mean and standard deviation of 8.0 ± 5.4 for the patient healthcare questionnaire-9 score (PHQ-9). Furthermore, 37.4% of HIV-positive pregnant women had a severe anxiety disorder, with a mean and standard deviation of 5.9 ± 4.6 for the Generalized Anxiety Disorder (GAD-7) score. Both of these scores were significantly higher than the scores for HIV-negative pregnant women (p = 0.000) [109].

Individuals who have been stigmatized for years because of their HIV status and carry the burden of a chronic virus are hesitant to seek medical care during the COVID-19 pandemic. The fear of contracting another more severe virus has made some people reluctant to even go to pharmacies to collect their antiretroviral therapy (ART) [110]. The physical distancing guidelines during the COVID-19 pandemic can increase the isolation experienced by older individuals with HIV who already face high levels of isolation [111]. Although there is no clear evidence that people living with HIV are at a higher risk of contracting COVID-19, they may still fear contracting it, which could lead to delayed medical care. Depression is a significant mental health concern among people living with HIV, and it can act as an additional barrier to accessing care [112]. The COVID-19 pandemic is a significant stressor and is likely to exacerbate the already high prevalence of mental health issues among people living with HIV [113]. Access to care has been significantly impeded by national lockdowns.

General and specific management of COVID-19 among PLWH

The treatment aspects of managing of COVID-19 patients along with HIV aren’t altered significantly, although, the chances of poor outcomes are heightened in these patients.

Hand washing, usage of N95 masks, and observing physical distances should be followed. Along with the COVID-19 vaccine, vaccination for influenza should also be administered. Patients who are on prophylactic medications for opportunistic infections of HIV should be continued. As there are no interactions of remdesivir with ART medications, they can be safely given with no dosage modifications. Extremely debilitated COVID-19 patients on ventilators can be given ART therapy either by crushing the tablets or liquid forms. Dexamethasone is usually prescribed for 10 days for COVID-19 infection. Hence, even though, it can interact with ART drugs by inducing hepatic enzymes, it can be safely administered due to the short course [114].

Public health organizations' guidelines recommend immediate initiation of ART in persons with newly diagnosed HIV infection [115]. Low CD4 count and high viral load increase the risk of progression to AIDS among COVID-19 patients. Therefore, CD4 count and viral load need to be monitored every three to six months. COVID-19 patients with HIV, who don’t require hospitalization can be given drugs such as ritonavir-boosted nirmatrelvir, intravenous remdesivir, and molnupiravir. Tocilizumab and dexamethasone are the drugs indicated in severe COVID-19 hospitalized patients, who are at risk for severe secondary infection. However, the efficacy and safety of these drugs in advanced HIV patients have not been studied [116].

Impact of the COVID-19 pandemic on HIV healthcare

It has been reported that there has been a decline in adherence to antiretroviral therapy (ART) among people living with HIV (PLHIV) due to the COVID-19 pandemic. However, there was no impact on viral suppression. Non-HIV-directed care challenges such as depression, anxiety, substance abuse, stigma, and discrimination were also observed. In-person visits and clinical follow-up services have been severely affected by the pandemic, although telemedicine has helped to alleviate the situation. The WHO reported that 36 countries had documented widespread disruption in ART services affecting 11.5 million people, and 24 countries suffered from inadequate ART supply impacting 8.3 million people receiving ART. The predicting factors for missing ART stock refill included but were not limited to fear of COVID-19, transport disruption, reduced income, unaffordability of traveling to healthcare facilities, and limited access to face masks. Other factors mentioned included healthcare accessibility disruption, missed clinical appointments, PTSD, and younger age. Pharmaceutical companies also faced difficulties in international shipping due to border restrictions, transportation delays, increased lead times, and increasing costs, leading to global ART disruptions [117].

A significant impact on the HIV healthcare system was reported in China, which led to a 49.0% decrease in reported HIV screening tests compared to expected data. Of those who tested positive during HIV screening, almost 60% received confirmatory tests, and only 80% of confirmed cases were registered in the healthcare system. Most of the newly diagnosed PLWH (People Living with HIV) did not receive timely health care services. During the COVID-19 pandemic, hospital-based screening resulted in 79.6% of all reported screening tests in 2019 for Jiangsu. However, hospitals had to shut down clinical services or shift their healthcare resources to complement COVID-19 control efforts during the pandemic. Consequently, HIV-related services were halted, decreasing the chances for people to check their HIV status. People also shied away from getting routine HIV testing, especially in hospitals during the pandemic period, as they feared facing COVID-19-related stigma and discrimination. Reduced sexual risk behaviors during the pandemic period also potentially led to decreased HIV testing rates. The pandemic also challenged the timely initiation of ART for newly diagnosed PLWH [118].

The COVID-19 pandemic was also observed to have a profound effect on the HIV pandemic with the percentage of positive tests, consultations, and new enrollments in HIV care in 44 countries on four continents between January and August 2020. Despite implementing several mitigation strategies, the pandemic had a significant impact on HIV care services. The number of people living with HIV who received antiretroviral therapy (ART) remained stable, but there was a reduction in HIV testing in 16 out of 19 countries and a decline in ART initiation in 28 out of 29 countries. The study used secondary data that may have been affected by incomplete or inaccurate information. It also did not analyze the impact of COVID-19 on access to condoms, post-exposure prophylaxis, and pre-exposure prophylaxis among populations at risk for HIV acquisition. However, the study acknowledged that client-centered strategies, such as HIV self-testing, multiple months’ dispensation of antiretroviral, community-led services, and alternative drug delivery, helped mitigate the negative effects of the pandemic. The study warned that COVID-19's disruption of health systems will have long-term consequences, including an increase in new infections, a resurgence in AIDS rates and mortality, and difficulty in accessing laboratory testing for STIs (sexually transmitted infections), especially HIV. The study also suggested that alternative options for health care provision, such as telemedicine, home-based appointments, and home delivery of drugs, could improve retention in care. Healthcare providers should build on the lessons learned to improve mitigation strategies and provide the best possible prevention and care services to clients [119].

Other discerning factors found in other studies included but were not limited to the challenge of locating patients who failed to follow up and getting them to respond to questionnaires. This difficulty may have been due to the patients being hard to reach, compounded by the pandemic's impact on research participation. Many of the patients initially considered to have follow-up problems had been misclassified, indicating a need for improved patient monitoring and registration systems. Patients who failed to follow up were found to have lower levels of education, unemployment, low income, and difficulties in maintaining clinical follow-up. There were more women in the group of patients who failed to follow up, which could have been due to the social inequalities that women face in Mexico. Additionally, the group of patients who failed to follow up reported a higher proportion of anxiety symptoms. The most frequently reported reason for failure to follow up was related to work obligations. Communication problems with the institution and lack of information about procedures were also reported as reasons for non-compliance. The study also highlights the need for psychoeducation in people living with HIV to ensure adequate medical follow-up behavior [120].

The impact of the COVID-19 pandemic on retention and engagement in HIV services also came under observation where the COVID-19 pandemic was opined to have had a varying impact on people living with HIV (PLWH) in the US, While studies from the southern region found minimal negative impacts on retention or engagement in healthcare services and medication adherence, studies from the western, northeast, and mid-west regions showed some impacts. Disparities were identified by HIV status, race/ethnicity, age, income, housing status, and access to video telehealth, and were found in studies from all regions except the northeast. Some studies compared pre-pandemic viral suppression rates to time periods during the pandemic, with one finding a significant increase in the odds of viral non-suppression after the initiation of ‘shelter-in-place’ orders within PWH who utilize a safety-net clinic. It was also mentioned that changes in viral suppression rates, participant-reported missed or canceled visits, and challenges accessing ART, in addition to data on HIV transmission rates, were important to fully understand the impacts of the pandemic on the long-term health of PLWH. Several studies highlighted that specific communities, including those experiencing homelessness, with substance use disorders, who are of minority race/ethnicity, MHSM, or who use public insurance or lost health insurance, have suffered deeply from interruptions in services pertaining to HIV during the pandemic. Adaptations in care models for PLWH, including telemedicine, messaging applications, and mail-order pharmacy, have been critical interventions to ensure continuity of care during the pandemic. However, what’s important to note is the fact that severe disparities exist in which communities have access to the resources needed to utilize telemedicine. Lack of human connection, lack of knowledge on how to use telemedicine, and increased risk for disclosure of personal information also support the idea that telemedicine is not a suitable replacement for in-person visits. [121-129].

In a large southeastern comprehensive care clinic in the United States, patients in care were evaluated between January 2017 and July 2020 in a study. According to the study, during the first wave of the COVID-19 pandemic, there was a decrease in outpatient medical encounters and new patient appointments, while the number of mental health encounters increased in a large southeastern HIV clinic. Additionally, it was found that the proportion of patients without a documented HIV-1 RNA level also increased during the study period. The decrease in outpatient encounters may have been attributed to clinic closures or interruptions in care due to the pandemic. Patients with appointments rescheduled were more likely to have uncontrolled HIV or be at higher risk of being lost to follow-up, which suggests that providers prioritized patients who needed more frequent and direct contact. Mental health encounters increased during the study period, likely due to the pandemic-related stressors experienced by people with HIV [130].

A study conducted in Africa suggested that a six-month interruption in antiretroviral therapy (ART) supplies for 50% of people resulted in an approximately 1.63 times increase in HIV-related deaths over a year. This increase in sub-Saharan Africa alone would amount to a median of 296,000 excess HIV deaths over this period. The study showed that the CD4 lymphocyte cell count recovery which takes years to achieve on ART was rapidly lost after viral replication resumed in the absence of ART. The interruption of condom supplies, pre-exposure prophylaxis (PrEP), and peer education made populations more susceptible to increases in HIV incidence, although physical distancing measures lead to reductions in risky sexual behavior. The study did not specifically consider service interruption for key populations such as homosexual men and female sex workers as well as other MHSM, but given the levels of stigma around these populations, they could be particularly susceptible to interruptions in services. Although some uncertainty existed around the magnitude of the effect that program disruptions would have on the HIV epidemic, the interruption of ART was generally agreed to be the greatest threat to HIV mortality and incidence. The study concluded that any loss to the functioning of HIV programs would lead to adverse health consequences for many people [131].

Differences and similarities between HIV and SARS-CoV-2 epidemics

Differences

In the early 1980s, AIDS spread like wildfire throughout the world while the government tried to quell the panic in the public by broadcasting that it would not spread to the general population. Today, the emergence of COVID-19 as a pandemic is by far the biggest health-scare that we have seen in this century, perhaps even bigger than AIDS itself. There is a constant bombardment of messages in the media about how easily transmissible it is which has rightfully led to a fear of its imminent global spread in a matter of weeks, if not months. This new coronavirus is scarier as it is more contagious than expected, does not spare young and healthy individuals, and the dreaded respiratory failure has claimed many lives. The panic was enhanced by horror stories and misinformation shared on social media, especially, at the beginning of the pandemic [132].

It is an established fact that testing is critical in limiting the number of new infections in SARS-CoV-2 and HIV. Despite the huge demand for testing and subsequent shortage of test kits, routine testing for SARS-CoV-2 has been consistently high. In contrast, despite recommendations from the CDC for routine HIV testing of everyone between the ages of 13 and 64, there seems to be no urgency among providers to order the tests. Patients do not prefer to be tested, mainly due to the stigma associated with HIV. Another difference is the fact that HIV-1 cannot be eliminated once it infects the host, which constitutes a major roadblock in the treatment path, this issue is not faced with SARS-CoV-2. The rates of evolution of the two viruses are different as well, AIDS with at a rate of more than 1% a year and SARS-CoV-2 resting at less than 1 % [133].

Similarities

Both viruses share a number of epidemiological characteristics, such as having emerged as a zoonosis, with animal reservoirs, bats in the case of SARS-CoV-2 and non-human primates in the case of HIV-1, asymptomatic propagation, a disproportionate impact on people of color, gender or age and the necessity for quick diagnostic testing. Both illnesses are characterized by a lymphokine storm, which is related to the viral load, and lymphopenia is a prognostic parameter [133,134].

In AIDS, it is related to chronicity of the illness, with IL-6 being a major contributor and related to increased morbidity including cardiovascular disease, cancer, and others, and mortality in patients with controlled HIV replication. In SARS-CoV-2, the response is more acute and manifests clinically. An exaggerated inflammatory response occurs following an influx of inflammatory cells from the bloodstream. This is why therapies aim to block cytokines. The cytokine storm also leads to the infamous acute respiratory distress syndrome or multiple-organ dysfunction that is fatal in the susceptible population [134].

Both diseases require robust contact tracing measures in order to reduce transmission and behavior change in terms of protective measures i.e., masks vs. condoms. Both have had serious economic and social consequences.

Into the future

In the years to come, it will be vital to know how to handle patients and cadavers with SARS-CoV-2 or other such infections. During COVID-19, a triage approach and an understanding that all cadavers likely had the infection provided some solution. At the end of it all, the long-term effects of a pandemic really depend on how long it remains active. For example, SARS-1 and influenza never became pandemics, and HIV on the other hand remains active to date. How vaccines alter the course of a pandemic is obvious but we already know that the development of a vaccine against HIV seems unlikely. However, hope remains that COVID-19 vaccines will triumph in the end [134].

With the progress of the COVID-19 pandemic, several challenges related to its identification and global spread will be faced. Epidemiologically, there is a need to closely monitor the trends of COVID-19 impact worldwide, particularly in countries with substantial risk and burden of HIV to understand short and long-term consequences deeply. The research regarding diagnostic and therapeutic modalities should be conducted at a brisk pace to advance our knowledge to prepare well for future waves. It is imperative to understand the threat and curtail the disease to mitigate morbidity and mortality. Some major studies reviewed in this article have been outlined in Table 1 to assist physicians and health care providers to better cater to the disease with limited but sound scientific evidence.

**Table 1:** Summarized table of studies showing the correlation between COVID-19 and HIV COVID-19: Coronavirus disease 2019; CRP: C Reactive Protein; HbA1c: Hemoglobin A1c; PLWH: People living with HIV

Reference	Study Design	Date of Publication	Findings and Results	Conclusion
Mnguni et al. [60]	Retrospective Observational study	28 Feb 2023	Study in South Africa. The median age of PLWH with SARS-CoV-2 infection- 46 Male sex- 29.1% Median CD4 count- 267 Patients admitted- 255 Patients discharged- 195 Patients died- 60 Patients on ART-169 (88%) Patients on ART having AIDS-73 (28%) Smoking risk ratio- 2.86 Neutrophilia risk ratio-1.024 HbA1c risk ratio- 1.01	Multiple factors such as CRP, neutrophilia, and HbA1c play a significant role in the outcome of the disease and are the major cause of mortality.
Moreno-Torres et al. [63]	Retrospective observational analysis of data from the national registry	1 Jan 2023	Study in Spain. The number of adults hospitalized with covid 19- 117694. HIV positive-234 (0.2%) Patients on ART- >95% Mean age of HIV positive patients- 53.2 years Mean age of HIV negative patients- 66.5 years Rate of baseline liver disease in PLWH- 27.4% Rate of baseline liver disease in HIV negative people- 4.4%. In- hospital mortality of HIV positive patients-9.4%. In-hospital mortality of HIV negative patients-4.4%	In this study, HIV-positive patients admitted to the hospital had better survival and better recovery rate than HIV-negative patients. Advanced liver disease was the major predictor of death in HIV-positive hospitalized patients suffering from HIV.
Facciola et al. [81]	Cross-sectional study	17 Oct 2022	The study was conducted in Italy. Number of HIV-positive people on ART- 84 (85.1% were men). The number of HIV-negative healthcare workers (control group) - 64 (80.5% were men). Both groups received two doses of COVID-19 vaccination. In HIV positive group:- Number of individuals vaccinated with BNT162b2 COVID-19 mRNA (Pfizer/BioNtech)- 84.3% Number of individuals vaccinated withChAdOx1-nCoV-19 (AstraZeneca)-8.4% Number of individuals vaccinated with NIAID’s mRNA-1273 (Moderna)- 3.6%	This study shows the high efficacy of the mRNA vaccine in HIV-positive people.
Nkosi et al. [38]	Longitudinal observational cohort study	26 July 2022	Participants are categorized as: HIV seronegative- HIV negative, Suppressed-viral load below 50 copies/ml, and Viremic- viral load >1000 copies/ ml. Absolute CD4+ count correlated positively with Sar-Cov-2 specific CD4+ (r= 0.5, p= 0.03) and CD8+ (r= 0.5, p= 0.001) T cell response. T cell activation was negatively correlated with CD4+ T cell response (CD4 r=0.7, p=0.04) Viremic PLWH had narrow breadth of SARS-CoV-2-specific CD8+ (p=0.039) and CD4+ T cell responses (p=0.033)	Impaired T cell response is seen in people with unsuppressed HIV infection. Viremic PLWH have poor cross-recognition potential and have a narrow breath of SARS-CoV-2 specific CD8+ and CD4+ T cell responses.
Bertagnolio et al. [49]	Descriptive statistics and regression analysis	9 July 2022	Analysis of data from the WHO clinical platform for COVID-19 from 38 countries: PLWH- 16995 PLWH from Africa- 16283 (96.0%) Female patients- 10603 (62.9%) Males patients- 6271(37.1%) Number of hospital admissions- 6339 (38.3%) Deaths in hospital- 3913 (24.3%) People with HIV showed a 15% increased risk of severe presentation of COVID-19 and 38% more likely to die in a hospital.	HIV is an independent risk factor for severe COVID-19 at admission and in-hospital mortality.
Coburn et al. [77]	Longitudinal cohort study	1 June 2022	A study conducted in the United States. Sample size- 109599 HIV positive-31840 HIV negative-77759 Rate of breakthrough COVID-19 infection in PLWH- 44/1000 person-years Rate of breakthrough COVID-19 infection in HIV-negative people- 31/1000 person-years.	Breakthrough infection risk was found to be higher in PLWH as compared to HIV-negative people
Ade-Ojo et al. [109]	Cross-sectional study	16 April 2022	A study conducted in Southwest Nigeria. Number of pregnant HIV Positive women - 99. Number of pregnant females in the control group- 99	Anxiety and depression were significantly more common in HIV-positive pregnant females as compared to HIV-negative females during the COVID-19 pandemic.
Govere-Hwenje et al. [80]	Cross-sectional study	12 April 2022	A study conducted in South Africa. Number of participants- 213 Number of females- 153 (72%) Number of participants willing to accept future vaccines- 121 (57%) Number of participants unsure about future vaccination- 46 (22%) Number of patients not willing to accept future vaccination-45 (21%) Number of patients having fear of side effects- 42 (20%)	Over half of the PLWH were unsure or did not intend to be vaccinated in the future with further doses of COVID-19. This might be due to mistrust or false information among people regarding the safety of vaccination.
Rick et al. [119]	Retrospective cohort study	23 Feb 2022	Data collected from 44 countries in four continents (Asia, Latin America and the Caribbean, Europe, and Africa) indicating the impact of COVID-19 on HIV testing and HIV care during the pandemic suggesting the reduction in the new enrollments and failed mitigation strategies	The healthcare system must be strengthened during the pandemic so that no individual is spared from gaining health facilities.
Portillo et al. [76]	Prospective Cohort Study	10 Feb 2022	Number of individuals- 131. Mean age- 54 Males- 70%. Median baseline CD4 T cells-602cells/mm^3. Median nadir CD4 cells- 223 cells/mm^3 After vaccination, anti-RBD antibodies were found on day 30, day 60, and 6 months after the first dose. After 30 days of the first dose, HIV-1 RNA data were collected from 128 patients, out of which 19 patients (14.8%) had detectable HIV-1 RNA data were collected 30 days after the second dose from 124 patients, of which 15 had detectable HIV-1 RNA. Data were collected six months after the first dose from 83 patients, out of which 8 (9.6 %) had detectable HIV-1 RNA.	Appropriate RBD antibody response was elicited in all the patients after 2 doses of mRNA vaccination in HIV-positive individuals, with a minor impact on HIV-1 RNA levels after 6 months.
Danwang et al. [50]	Systemic review and meta-analysis	14 Jan 2022	Data were collected from 44 studies. Information was collected from 38,971,065 patients suffering from COVID-19. In unadjusted odd ratio- HIV positive individuals have higher chances of hospital admission- 1.49 In adjusted for age and sex, HIV was associated with a higher risk of death with a hazard ratio of 1.76	HIV seems to be an independent risk factor for increased risk of hospitalization and mortality in people suffering from COVID-19
Park et al. [43]	Cohort study	1 Jan 2022	Veterans Cohort Aging Study PLWH- 30,948. 189 patients positive for COVID. People without HIV- 76,618. 380 patients positive for COVID. Adjusted Odds Ratio- 1.04 (95% CI, 0.85-1.26)	HIV-positive status did not increase the risk of COVID-19 infection and severity. The risk of COVID-19 infection was similar among all ethnicities.
Levy et al. [78]	Prospective case-control Study	27 Dec 2021	After administration of BNT162b2 mRNA vaccine:- PLWH (cases)- 143 Healthy health care workers (controls)-261 In HIV positive individuals Anti- RBD antibodies after the second dose appeared in 139/141(98%) at a median of 18 days In Health care workers Anti- RBD antibodies appeared in 258/261 (98.9%) after a median of 26 days. HIV viral load increased in 2% of patients from <40 copies to <100 copies. Decrease in the CD4 count from 700 cells/mm^3 (95 % CI 648-757) to 633 cells/mm^3 (95% CI 588-683)	BNT162b2 mRNA vaccine appeared to be safe in PLWH with unsuppressed CD4 count and suppressed viral load on the ART regimen.
Yang et al. [47]	Population-level Cohort Study	8 Nov 2021	Study in United States of America Number of adult COVID-19 cases- 1436622 among these, HIV infected individuals- 13170 COVID-19 related deaths- 26130 among these deaths, HIV positive people- 445 Adjusted odd ratio of COVID-19 deaths and hospitalization in people with HIV- (1.29 (95 % CI, 1.16-1.44)) Odd ratio in mild to moderate COVID-19- (0.61 (0.59-0.64))	PLWH with low CD4+ count was associated with a higher risk of COVID-19 adverse effects.
Geretti et al. [61]	Prospective observational study	5 Oct 2021	Study Conducted in United Kingdom Number of patients- 47529 Confirmed HIV patients- 122 (0.26%) The cumulative 28 days mortality ratio PLWH- 26.7% The cumulative 28 days mortality ratio in HIV negative group- 32.1%	28 days mortality was higher in HIV-positive patients than HIV-negative patients.
Huang et al. [42]	Population-based cohort study	5 Oct 2021	Study in Wuhan Number of PLWH - 6001 COVID-19 positive- 35 (0.58% (95% CI, 0.42% - 0.81%)) Severe illness among COVID-19 patients- 15 (42.86 %) Number of Deaths- 2 People who discontinued ART- 197. Out of which 4 suffered from COVID-19.	Patients >50 years of age and people who have discontinued ART had a higher risk of COVID-19 infection. PLWH need to be supplied with antiretroviral drugs during the COVID-19 pandemic. The study found that cases with high viral load had fewer levels of IgM and IgG levels and those with low viral load had higher levels of IgM and IgG antibodies.
Boulle et al. [46]	A population cohort study	1 Oct 2021	Study in South Africa Total number of patients- 3460932 HIV positive patients- 16% Number of patients diagnosed with COVID-19 infection- 22308 Of whom, number of patients died- 625 Hazard ratio of HIV associated COVID-19 mortality – 2.14 (95% CI, 1.70-2.70) Standardized Mortality rate (SMR) for HIV associated COVID-19 death- 2.39 (95% CI, 1.96-2.86)	HIV and tuberculosis were independently associated with increased COVID-19 mortality. Deaths due to COVID-19 were associated with male sex, diabetes, increasing age, hypertension, and chronic kidney disease more often than HIV.
Nault et al. [75]	Observational Case-Control study	13 Aug 2021	Study in Montreal Number of participants- 121 Mean control group- 20 health care workers Number of patients showing anti COVID-19 antibodies in PLWH- 11 HIV-positive individuals with CD4 count <250 showed a lower response to anti-RBD IgG antibody (p < 0.0001)	Similar anti-RBG IgG antibodies response was seen in the control group and HIV-positive individuals with CD4 count >250 cells/mm^3^.
Shi et al. [118]	Observational Database Analysis	7 Aug 2021	Study conducted in Jiangsu, China, during the COVID-19 pandemic. HIV testing decreased by- 49%. Expected new HIV cases- 1555. Actual newly diagnosed HIV cases were 980 (63%) CD4 testing received by - 49.5% of patients initiated on ART- 91.6%.	COVID-19 had a negative impact on the healthcare system in Jiangsu, China. The COVID-19 pandemic lead to the delay in the diagnosis and commencement of ART regimens in HIV patients.
Calza et al. [52]	Observational Case series	April 2021	Study done in Northern Italy. Sample size- 26 HIV-infected patients Number of men- 19. Median age- 54 years Number of patients suffering from other comorbidities- 73% Number of hospitalizations- 5 No ICU admissions and deaths occurred.	PLWH suffering from COVID-19 had a similar clinical presentation as that of the general population and these people are frequently associated with other chronic comorbidities.
Mondi et al. [56]	Observational Case Series	March 2021	The study included five patients. CD4 count >350 cells/mm was found in 3 of them. Specific T cell response and IgG production were elicited in all patients. Patients showed a high degree of cytokine production and immune activation.	The study did not find any correlation between the increased risk of COVID-19 and HIV coinfection.
Tesoriero et al. [48]	Observational Cohort Study	1 Feb 2021	Study in New York People living with HIV-2988. PLWH hospitalized- 896 Number of Deaths - 207	PLWH had a poorer prognosis and higher rate of hospitalization due to COVID-19 than people without HIV infection.
Bhaskaran et al. [30]	Population-based retrospective cohort study	8 Jan 2021	Study done in the UK using the OpenSAFELY platform. Sample size- 17282905 HIV positive- 27480 Deaths due to COVID-19- 14882 Deaths in HIV positive people suffering from COVID-19- 25 Hazard ratio (HR) after adjusting for age and sex-2.90. HR after adjusting for deprivation, ethnicity, smoking, and obesity-2.59. HR in black ethnicity was found to be 4.31 and in non-black as 1.84.	PLWH had a greater risk of COVID-19 mortality. Other factors such as obesity, male sex, deprivation, black ethnicity and smoking elevated the risk of mortality.
Sachdev et al. [44]	Descriptive statistics of the Department of public health database	1 Jan 2021	Study in San Francisco. PLWH- 4252 COVID-19 positive- 193(4.5%) The average age of people infected with HIV/COVID-19 - 48 years. The percentage of ethnicities were: Whites- 38.9%, Latinx- 38.3%, African American- 11.9%, and Asian – 6.7 %. Out of which 91.2% were men, and 54.6% of coinfected people were housed.	The severity of COVID-19 illness remained almost the same in PLWH and people living without HIV with higher rates of infection in people with congregate living situations.
Sigel et al. [18]	Observational cohort study	31 Dec 2020	Study in New York COVID-19 positive hospitalized PLWH (sample size) - 88 Hospitalized PLWH requiring mechanical ventilation- 18% Hospitalized PLWH died- 21% Non-HIV people requiring ventilation- 23% Non-HIV people died due to COVID-19- 20%	No significant difference was found in the adverse outcomes of the PLWH and non-HIV group suffering from COVID-19.
Elfiky et al. [68]	A molecular docking study	1 Oct 2020	Phylogenetic analysis of the RNA dependant RNA polymerase (RdRps) of 7 Human Coronaviruses (HCoVs) (229E, NL63, HKU1, OC43, MERS, SARS, and Wuhan SARS-CoV-2) was performed. Drugs binding to SARS-CoV-2 RdRp with binding energy (kcal/mol):- Galidesivir-−7.0, Remdesivir-−7.6 Tenofovir- −6.9 Sofosbuvir-−7.5 Ribavirin−7.8 These drugs contradict the polymerase function. These 5 drugs can bind to SARS-CoV-2 RdRp, with binding energies comparable to those of the native nucleotides.	The study concluded that Tenofovir, Ribavirin, Remdesivir, Sofosbuvir, and Galidesivir showed efficacy against RNA Dependent RNA polymerase enzyme of the SARS-CoV-2 strain.
Inciarte et al. [39]	Cohort Prospective observational study	1 Oct 2020	Study conducted in Spain Sample size (PLWH)- 5683 COVID-19-positive- 53(100%) Symptoms: Cough- 87%, Fever-82% Hospital Admissions- 49% Severe disease- 14% ICU admission- 8% Mortality- 4%	The clinical characteristics, risk factors, or other symptoms of COVID-19 in PLWH did not differ from the remaining control group of the HIV cohort. The incidence of COVID-19 was found to be low in PLWH.
Charre et al. [41]	Retrospective analysis of laboratory database	1 Oct 2020	Study in France. Number of patients (sample size)-19113 HIV infected-77 Pre-exposure prophylaxis users- 27 These patients were assessed for SARS-CoV-2 PCR assay: Corrected COVID-19 attack rate in HIV infected patients- 0.31- 0.38% Pre-exposure prophylaxis user- 0.38/0.42% Attack rate in the general population- 0.24%	The risk of COVID-19 infection appeared similar in PLWH, patients with pre-exposure prophylaxis, and the general population.
Karmen-Tuohy et al. [56]	Observational Cohort study	1 Sept 2020	Study conducted in New York. HIV-positive patients- 21 non-HIV patients- 42 Inflammatory markers were found to be similar. HIV-positive individuals had a higher rate of hospital admission.	The clinical presentation, hospital course and outcome of the COVID-19 disease in HIV-positive patients are similar to the non-HIV individuals.
Miyashita et al. [59]	Observational cohort study	23 Aug 2020	Study done in New York. COVID-19 patients (sample size)- 8912 HIV positive-161 (1.8%) Younger patients less than 50 years of age had a higher risk of intubation (relative risk- 2.97 (95% CI 1.29-6.84)) and death (relative risk- 4.46 {95% CI 1.43-13.3})	There is a higher risk of intubation and death in younger COVID-19 patients suffering from HIV than in non-HIV individuals. HIV-infected patients had a higher prevalence of chronic diseases such as hypertension, diabetes, dyslipidemia, and heart failure.
Guo et al. [69].	Observational Cohort Study	10 Aug 2020	Study in Wuhan, China HIV positive patients investigated- 1701 Proportion of COVID-19 in PLWH- 0.6% (95% CI 0.2-1.0) Proportion of COVID-19 to the overall population in Wuhan- 0.6% Average CD4+ T lymphocyte count- >200 Average viral load- <20 copies Older PLWH had low CD4 cell count.	People with HIV/AIDS are at equal risk of infection as that of the general population. People with low CD4 counts must be given additional attention.
Del Amo et al. [40]	Cohort study	26 June 2020	Study conducted in Spain. HIV positive- 77590 COVID-19-positive- 236 Hospitalized- 151 ICU admission- 15 Deaths- 20 Risk of hospitalization among patients receiving different combinations of therapies:- 1. TAF/FTC= 20.3 (95% CI 15.2 – 26.7) 2.TDF/FTC- 10.5 (CI 5.6 – 17.9) 3. ABC/3TC= 23.4 (17.2-31.1) 4.Other regimens= 20.0 (CI 14.2 – 27.3)	Patients on TDF/FTC had a lower risk for COVID-19 and related hospitalization as compared to patients on other regimens.
Vizcarra et al. [70]	Observational prospective cohort study	28 May 2020	Study in Madrid Cases reported in the clinics: HIV infected individuals- 2873 Diagnosed with COVID- 51 Rate of infection- 1.8% (95% CI 1.3-2.3) Laboratory confirmed cases- 69% Suspected cases- 31% Mean age of HIV infected individuals suffering from COVID-19 19- 53.6 years Data of cases reported in the community of Madrid general population: COVID-19 cases- 269417 laboratory confirmed- 61577 Suspected- 207840 Overall covid infection rate- 4.02% Mean age of COVID-19 infection among the general population was more uniform. 63% of COVID-19 patients had at least one comorbidity compared to only 38% without covid (p= 0.00059). 73% of patients received tenofovir before COVID-19 diagnosis compared to 38% without COVID-19 (p= 0.0036). Critically ill- 12% deaths- 4%	The general population had a higher COVID-19 infection rate as compared to HIV-infected individuals. Clinical symptoms, analytical and radiological analysis of COVID-19 were similar in HIV-positive patients and in the general population. No specific antiretroviral drug has been found to increase the severity of COVID-19.
Blanco et al. [57]	Observational Case Series	15 April 2020	Study conducted in Spain. Number of hospital admissions due to COVID-19- 543 Number of ICU admissions- 62 (12%) Discharged- 208 (38%) HIV positive patients- 5 Virologically suppressed patients – 4 Patients required ICU care- 2	Management of patients co-infected with HIV should be improved. There might be a need for change in the anti-retroviral drugs depending upon the response of the patients.

## Conclusions

The review highlights that susceptibility to COVID-19 infection among PLWH is variable, as depicted in different studies, although various confounders, including comorbid hypertension and diabetes and age and ethnicity, can alter the result markedly. The relationship between HIV and COVID-19 remains debated, with conflicting results concerning vulnerability and severity of outcomes. Further research is needed to understand the interaction between the two viruses, considering factors such as disease stage, CD4 count, and antiretroviral treatment. COVID-19 has been treated with several antiviral drugs and protease inhibitors. Whereas COVID-19 vaccines have been deemed safe for PLWH with no specific allergies reported so far, nonetheless, low CD4+ cell counts may reduce vaccine immunity.

However, pandemics are different in how they spread around the world, how they affect people of different ages, and how they are tested. The COVID-19 pandemic has disrupted HIV treatment and care, causing fear, depression, and supply chain issues that result in ART shortages. Among various mitigation efforts, some worth mentioning include telemedicine, home calls, and drug delivery. People have said that both pandemics require care from different fields and strategies to meet the needs of the most vulnerable people. Contact tracing, protective measures, and addressing risk factors like age, weight, diabetes, and race are essential for prevention. The COVID-19 pandemic has particularly affected fragile HIV-positive individuals, disrupting HIV clinics and emphasizing the need for effective management of both diseases. The future of these pandemics will depend on how well vaccines and other measures to keep them under control work. Continued research and understanding of the complexities of HIV and COVID-19 interactions are crucial to supplying adequate support and care for affected populations, ultimately working towards reducing the burden of these global health crises.
